# Oral health status and access to oral healthcare among adult refugees in Egypt: a cross-sectional study

**DOI:** 10.1186/s12903-025-07541-7

**Published:** 2026-01-17

**Authors:** Dina Attia, Mona K. ElKashlan, Susan M. Saleh, May M. Adham

**Affiliations:** Department of Pediatric Dentistry and Dental Public Health, Faculty of Dentistry, Alexandria University, Alexandria, Egypt

**Keywords:** Oral health, DMFT index, CPI index, Healthcare access, Refugees, Egypt

## Abstract

**Background:**

Refugees oral health remains underexplored despite high disease prevalence. This study assessed oral health, access to care, and factors associated with dental caries and periodontal disease among refugees in Alexandria, Egypt.

**Methods:**

A cross-sectional study was conducted at Caritas office from June-September 2024. Arabic-speaking refugees aged ≥18 years, residing in Alexandria for ≥6 months were included. Data were collected via a questionnaire and oral examination using Silness and Loe Plaque Index, Decayed, Missing, and Filled Teeth (DMFT) index, and Community Periodontal Index (CPI). Negative binomial regression identified factors associated with DMFT, and binary logistic regression identified factors for periodontal pockets.

**Results:**

A total of 510 refugees participated (64.1% females, mean age 46.7 ± 14.2 years, 79.0% Sudanese). Mean DMFT was 9.25 ± 5.82 (DT = 5.01, MT = 3.34, FT = 1.02), and mean Plaque Index was 1.49 ± 0.68. Gingivitis was found in 64.51% and periodontal pockets in 12.16%. In multivariable analysis, increased caries prevalence was associated with age (PRR = 1.16, 95% CI: 1.03–2.34), education (illiterate vs. university educated, PRR = 1.32, 95% CI: 1.08–3.49; secondary vs. university educated, PRR = 1.19, 95% CI: 1.05–4.32), low income (PRR = 1.06, 95% CI: 1.01–1.63), sugary snacks (PRR = 1.39, 95% CI: 1.29–3.51), and Plaque Index (PRR = 1.46, 95% CI: 1.20–3.53). Shorter residence (≤5 years) (PRR = 0.90, 95% CI: 0.71–0.93) and access to oral healthcare (PRR = 0.88, 95% CI: 0.56–0.98) were associated with lower DMFT count. Periodontal pockets were associated with male gender (AOR = 1.87, 95% CI: 1.34–5.17), older age (AOR = 4.48, 95% CI: 2.68–10.62), education (illiterate AOR = 5.15, 95% CI: 1.28–17.64; secondary AOR = 2.61, 95% CI: 1.92–14.32), sugary snacks (AOR = 2.47, 95% CI: 1.20–6.09), smoking (AOR = 3.18, 95% CI: 1.56–9.12), Plaque Index (AOR = 3.60, 95% CI: 1.92–8.30), and DMFT (AOR = 2.78, 95% CI: 1.09–6.98).

**Conclusions:**

Refugees had a high burden of oral disease, with significant sociodemographic, behavioral, and clinical factors. Tailored oral health promotion programs are needed for this vulnerable group.

## Introduction

By the end of 2024, the global refugee crisis reached unprecedented levels, with more than 123 million people displaced worldwide due to oppression, conflict, and human rights violations. Among them, 36.4 million are officially recognized as refugees, with approximately 76% hosted by low- and middle-income countries [1, 2]. International law defines a refugee as "a person who has fled from and/or cannot return to his or her country due to a well-founded fear of persecution, including war or civil conflict [3]. Throughout their journey, refugees face great health challenges from displacement to resettlement, where they are exposed to trauma, disease, and elevated stress levels, which severely affect their physical, mental, and emotional well-being [4].

Access to healthcare is a critical challenge for refugees, where factors such as legal status, unfamiliarity with healthcare systems, unemployment, and migration-related trauma make it difficult to obtain appropriate medical care. These barriers exacerbate health risks and further harm their overall well-being, highlighting the urgent need for healthcare solutions that are comprehensive and specifically tailored to meet the specific needs of this vulnerable group [5, 6].

Egypt ranks among the top five countries with the largest number of urban refugees in the world, and is one of the only two African countries that do not require that refugees reside in camps [7]

In Egypt, migrants and refugees have equal rights to access primary healthcare services like Egyptian citizens; however, the Egyptian healthcare system is struggling to meet the needs of this growing population, especially with the already burdened socio-economic situation [8]. In 2022, International Organization for Migration (IOM) reported that the current number of international migrants residing in Egypt was 9,012,582, constituting 8.7% of the Egyptian population (103,655,989) [9]. As for refugees, according to the United Nations High Commissioner for Refugees (UNHCR) report, Egypt received the second-highest number of new asylum applications globally in the first half of 2024 [10]. By the end of June 2025, there were more than one million officially registered refugees from more than 59 countries with the Sudanese refugee population being the top nationality with 73% of the refugee population, followed by Syrians at 13.5%. Most refugees are situated in urban areas, in Cairo, Alexandria, and Sharkia [11, 12] About 15% of the migrant population in Egypt (1–1.3.3 million) are classified as “vulnerable” or “people of concern”, in need of a direct assistance [9]. The term 'people of concern' specifically refers to individuals under the UNHCR's mandate who require international protection, such as refugees, asylum-seekers, stateless persons, and returnees. The 'vulnerable' classification further identifies individuals within these groups who face acute risks due to factors like extreme poverty, serious health conditions, or having experienced violence or torture [1]. This acute need is evidenced by the 2016 categorization of 90% of Syrian refugees in Egypt as severely vulnerable [13]. Furthermore, in 2022, the wider refugee population in Egypt was classified among the most vulnerable populations overall due to the worsening socio-economic conditions caused by inflation and increased cost of living [9].

While the general and mental health of refugees have been extensively studied [6, 14, 15], their oral health remains an underexplored area of research. This gap is critical because oral health is fundamental to overall well-being, impacting essential functions like nutrition and speech, and is linked to systemic health conditions. Moreover, access to dental care is a key determinant in maintaining sound oral health enabling preventive care and timely treatment of diseases [16]. To date, no study has specifically assessed oral health status and access to dental care among refugees residing in Egypt. However, a recent scoping review summarizing global evidence confirms that refugees consistently experience a higher prevalence of oral diseases compared to host country populations, with studies from various host nations including Germany, Canada, Australia, Jordan, and Iraq supporting this finding [17]. This review underscores that a high prevalence of dental caries and limited access to dental care are major challenges faced by refugees and asylum seekers worldwide [17]. Periodontal disease and dental caries are the two most prevalent oral diseases worldwide and are a major contributor to the global burden of chronic disease [18]. Effective prevention of these diseases depends on access to oral healthcare, periodic assessment of oral health and individual oral health behavior. Therefore, this research aimed to assess oral health status and access to oral health care of refugees in Alexandria, Egypt, to assess their needs and provide a basis for reporting their condition, where despite their substantial numbers, published reports on their health status are scarce. By establishing this baseline data, this can assist humanitarian stakeholders and host governments to develop and implement targeted intervention programs and allocate resources effectively.

## Materials and methods

### Study design

A cross-sectional study was conducted between June 2024 and September 2024 at the Caritas Egypt Refugees office in Alexandria. The Caritas Egypt Refugees office has served refugees coming from different places all over the world since 1977 through a variety of services, such as medical services, vocational training, monthly subsistence allowance, and emergency grants [19]. Participation was voluntary, using snowball and chain referral sampling techniques, which are recommended for sampling rare and difficult-to-reach populations [20].

Ethical approval was obtained from the Dental Research Ethics Committee, Faculty of Dentistry, Alexandria University (IORG0008839, 0930-06/2024), and approval from Caritas management. All participants gave their written informed consent before enrollment. This study was conducted in accordance with the Declaration of Helsinki.

### Participants

Inclusion criteria were: (1) refugees aged 18 years and older, (2) originally from Arabic-speaking countries, (3) residing in Alexandria, Egypt, for at least six months, and (4) agreed to participate in the study. Sample size was determined using a calculator for estimating population proportion (https://www.calculator.net/sample-size-calculator.html) based on assuming confidence level= 95% and margin of error= 5%. Because there was no information about the oral health status among refugees in Egypt, the sample size was calculated based on a prevalence of 50% (this value maximizes sample size and is assumed in the case where there is no information on the prevalence in the study area) [21]. The minimum required sample size was 385, which was increased to 500 to make up for non-response bias.

### Study measures

Data were collected through oral examination and an interview-based questionnaire. Dependent variables in this study were dental caries and periodontal disease. Independent variables included migration-related factors, dental visits, and access to primary oral healthcare. Sociodemographic characteristics and oral health-realted practices were considered potential confounders.

### Questionnaire

The questionnaire was developed based on the studies by Karnaki et al., and Marwaha et al. [22, 23]. The questionnaire consisted of 3 sections. The first section assessed the sociodemographic factors including sex (male, female), age, country of origin, marital status (married, unmarried), number of children, educational level (illiterate, secondary or less, or university and higher). The second section assessed migration-related factors including immigrating status (alone or with family), years of residence in Egypt (6 months-2 years, 2–5 years, >5 years), employment status (employed, searching for a job, not searching for a job), [24] and income per month (<3000 EGP, 3000-6000 EGP, >6000 EGP) [25]. The last section assessed oral health-related practices including toothbrushing frequency (at least once daily, few times per week, rarely/never), tools used for adjunctive teeth cleaning (dental floss, wooden or plastic toothpicks, Miswak, charcoal, salt, lemon, none), access to primary oral healthcare (yes, no), timing of last dental visit (<6 months, 6–12 months, > 1 year but less than 2 years, >2 but less than 5 years, > 5 years, never received dental care), and the reason of this visit (pain, treatment/follow-up, regular check-up), frequency of sugary snacking (at least once daily, few times per week, rarely/never), and cigarette smoking (yes, no). The questionnaire was first developed in English, translated into Arabic, and then back-translated to English to confirm accuracy, and six experts evaluated its content validity. The content validity of the questionnaire was evaluated using the content validity index, calculating item-level (I-CVI) and scale-level indices (S-CVI/UA and S-CVI/Ave) [26]. The I-CVI ranged from 0.821 to 1.00, the S-CVI/UA based on universal agreement was 0.864, and the average S-CVI (S-CVI/Ave) was 0.965. The questionnaire was pilot-tested with 30 refugees to ensure clarity. Data from the pilot test was excluded from the final analysis. The questions were asked in Arabic in a face-to-face interview.

### Oral examination

In this study, oral health status was evaluated using Silness and Loe Plaque index, Decayed Missing Filled Teeth (DMFT) index, and Community Periodontal Index (CPI) index. Dental examination was conducted by a single trained and calibrated dentist (DA). To assess intra-examiner reliability, 10% of the sample was re-examined after a 7-day interval. For these participants, only the data from the initial examination was used in the final analysis. The re-examinations yielded excellent agreement for all indices, with Kappa scores of 0.81 for plaque index, 0.88 for DMFT index, and 0.84 for CPI [27]. All refugees who met the inclusion criteria were examined while seated in a regular chair, using flat disposable dental mirrors and blunt-tipped dental probes using headlight for better illumination. Assessment was done following the World Health Organization's Oral Health Survey diagnostic criteria [28]. Oral hygiene status was assessed using the Silness and Loe Plaque index to assess the thickness of plaque at the gingival area of 6 index teeth (1.6, 1.2, 2.4, 3.6, 3.2 and 4.6). Each surface (buccal, lingual, mesial, distal) was scored based on the following criteria: 0 for no plaque; 1 for a film of plaque detectable only by running a probe across the tooth surface; 2 for moderate soft deposits visible to the naked eye, accumulating within the gingival pocket or on the tooth; and 3 for an abundance of soft matter filling the area between the gingival margin and the tooth surface. The scores from the four areas of the tooth were added and divided by four in order to give the mean for this tooth. Then, the scores of the examined teeth were added and divided by the number of examined teeth to get the Plaque Index value [29]. For caries assessment, the teeth were first cleaned with gauze to remove any soft debris, then visually examined and probed to identify cavities. White spot lesions were categorized as sound, whereas teeth with temporary fillings or restorations showing decay were marked as decayed. No radiographs were used in the examination. All erupted teeth were assessed and coded as D (cavitated), M (missing due to caries), or F (filled). DMFT was calculated by adding the scores of the 3 categories. Finally, the periodontal status was assessed using CPI index with WHO CPI probe (with a 0.5 mm ball tip and markings at 3.5 mm, 8.5 mm, and 11.5 mm). The whole mouth was divided into six sextants and periodontal health status was assessed on a scale ranging from 0 to 4 using the three components of the community periodontal index (CPI); bleeding on probing (CPI 1), presence of calculus (CPI 2) and pocket depth formation 4- to 5-mm deep (CPI 3), or a 6-mm or deeper pocket depth (CPI 4). CPI score 0 was given to participants with healthy gums and sextants were examined when they included at least two functioning teeth. Ten index teeth (1.7, 1.6, 1.1, 2.6, 2.7, 3.7, 3.6, 3.1, 4.6, 4.7) were examined by moving the WHO CPI probe slowly from the distal to the mesial surfaces along both the buccal and lingual sulci of each of the index teeth. No replacement tooth was examined if an index tooth was missing, however when all index teeth were missing from a sextant, all present teeth in that sextant were examined instead. The highest tooth score was, then, recorded as the score for a sextant and the highest CPI score for all examined teeth was recorded as the CPI score for each participant [28].

### Data analysis

Data analysis was performed using Statistical Package for the Social Sciences 29.0 (SPSS Inc., IBM). Continuous data were presented as mean and standard deviation, while categorical data were presented as frequency counts and percentages. To identify factors associated with dental caries experience, a negative binomial regression model was performed with DMFT values as the dependent variable. The model estimated prevalence rate ratios (PRRs) with their 95% confidence intervals (CIs) to quantify the association between various independent variables and the expected counts of caries experience. For the analysis of factors associated with periodontal pockets, a binary logistic regression model was used, where the dependent variable was dichotomized using CPI index scores (≤2 vs. ≥3). The model estimated adjusted odds ratio (AOR) along with their 95% CIs. Both univariate and multivariate analyses were conducted for each outcome. In the univariate analysis, the association of each independent variable with the dependent variable was assessed individually. Then, a multivariate model was constructed including all variables to determine their independent effects while adjusting for potential confounders. Prior to running the final multivariate models, multicollinearity between the independent variables was assessed using variance inflation factor (VIF), where VIF= 1/tolerance. No collinearity was detected, as all the tolerance values were > 0.2 and VIF values were < 5 [30]. Statistical significance was set at a p-value of ≤ 0.05.

## Results

Except for four individuals who declined to participate, all refugees asked to participate agreed, resulting in a total of 510 refugees who provided written informed consent. The sociodemographic characteristics, migration-related factors, and oral health-related behaviours of the participants are shown in Table 1. Most of the participants were females (64.12%), of Sudanese origin (79.02%), and with a mean ± SD age of 46.7 ± 14.2 years. Approximately three quarters of the refugees (76.67%) were married and had an average of 2.8 ± 2.1 children, with 72.75% having at least one child. Only 185 refugees (36.27%) had attained university or higher education. Almost 90% of the refugees immigrated with their family and the majority (78.04%) resided in Egypt 6 months-2 years ago. More than half the refugees (58.62%) reported an income of less than 3000 EGP monthly, while 52.7% were actively searching for employment. Regarding oral health-related behaviors, although 318 participants (62.35%) reported brushing at least once daily, 110 refugees (21.57%) rarely or never brushed their teeth. As for adjunctive cleaning tools, the majority (62.94%) reported using no adjunctive cleaning tools; among those who did, charcoal (13.33%) and miswak (10.98%) were the most commonly reported methods. In addition, the vast majority of participants (87.84%) were unable to access or utilize primary oral healthcare services. Dental visits were not so frequent, as 176 refugees (34.51%) last visited a dentist 1–2 years ago, with the primary reason for dental visits being pain (70.6%). Concerning sugary snacking, 306 refugees (60.00%) reported consuming it at least once daily, and majority of the sample (83.14%) were non smokers.

**Table 1:** Sample characteristics (*N*= 510)

Variables	Frequency, n (%)
Gender	
Male	183 (35.88)
Female	327 (64.12)
Age (mean ± SD)	46.7 ± 14.2
Country of origin	
Sudan	403 (79.02)
Syria	81 (15.88)
Iraq	26 (5.10)
Marital status	391 (76.67)
Married	
Unmarried	119 (23.33)
Number of children (mean ± SD)	2.8 ± 2.1
Having at least one child	371 (72.75)
Educational level	
Illiterate	72 (14.12)
Secondary or less	253 (49.61)
University and higher	185 (36.27)
Immigration status	
Alone	57 (11.18)
With family	453 (88.82)
Time in Egypt	
>6 months-2 years	398 (78.04)
2-5years	29 (5.69)
>5 years	83 (16.27)
Employment status	
Employed	109 (21.4)
Searching for a job	269 (52.7)
Not searching for a job	132 (25.9)
Income level	
<3000 EGP	299 (58.62)
3000-6000 EGP	193 (37.84)
>6000 EGP	18 (3.54)
Toothbrushing frequency	
At least once daily	318 (62.35)
Few times per week	82 (16.08)
Rarely/Never	110 (21.57)
Adjunctive dental cleaning	
Tooth floss	18 (3.53)
Wooden/Plastic sticks	43 (8.43)
Miswak	56 (10.98)
Charcoal	68 (13.33)
Salt	32 (6.27)
Lemon	19 (3.73)
None	321 (62.94)
Access to primary oral healthcare	
Yes	62 (12.16)
No	448 (87.84)
Dental care utilization	
<6 months	25 (4.90)
6 - 12 months	59 (11.57)
> 1 year but less than 2 years	176 (34.51)
>2 but less than 5 years	149 (29.22)
> 5 years	81 (15.88)
Never received dental care	20 (3.92)
Reason for last dental visit (*N*= 490)	
Pain	346 (70.61)
Treatment/follow-up	131 (26.73)
Regular checkup	13 (2.66)
Frequency of sugary snacking	
At least once daily	306 (60.00)
Few times per week	136 (26.67)
Rarely/Never	68 (13.33)
Cigarette smoking	
Yes	86 (16.86)
No	424 (83.14)

Oral health status of the participants is shown in Table 2. For dental caries, the mean DMFT score was 9.25 ± 5.82. This comprised primarily of decayed teeth (DT = 5.01 ± 3.78), followed by missing teeth (MT = 3.34 ± 4.26), while filled teeth (FT = 1.02 ± 2.03) represented the smallest component. The mean ± SD Plaque index score was 1.49 ± 0.68, most of the refugees (64.51%) showed gingivitis (CPI scores 1–2), while a smaller proportion (12.16%) had periodontitis (CPI scores 3–4).

**Table 2:** Oral health status of participants (*N*= 510)

Variable	Value
DMFT (mean ± SD)	9.25 ± 5.82
DT (mean ± SD)	5.01 ± 3.78
MT (mean ± SD)	3.34 ± 4.26
FT (mean ± SD)	1.02 ± 2.03
Plaque index (mean ± SD)	1.49 ± 0.68
CPI (n, %)	
0 (Healthy)	119 (23.33)
1–2 (Gingivits)	329 (64.51)
3–4 (Periodontitis)	62 (12.16)

Table 3 shows regression analysis for factors associated with dental caries. In the fully-adjusted model, several factors were significantly associated with higher expected counts of caries experience. For instance, an increase in age was associated with 16% higher caries experience (PRR= 1.16; 95% CI: 1.03–2.34.03.34). As for educational level, illiterate participants had 32% greater caries counts (PRR= 1.32; 95% CI: 1.08–3.49.08.49) and those with secondary education or less showed 19% higher counts (PRR= 1.19; 95% CI: 1.05–4.32.05.32) compared to university-educated individuals. Lower income was associated with 6% higher expected counts of caries experience (PRR= 1.06; 95% CI: 1.01–1.63.01.63). Consuming sugary snacks at least once daily was associated with a 1.39-fold greater prevalence of DMFT counts compared to those who consumed it irregularly (PRR= 1.39; 95% CI: 1.29–3.51.29.51). A higher Plaque index scores was associated with 46% increased counts (PRR= 1.46; 95% CI: 1.20–3.53.20.53). In contrast, refugees who had been in Egypt for 5 years or less had 10% less caries experience (PRR= 0.90, 95% CI: 0.71–0.93.71.93), and having access to primary oral healthcare was associated with 12% lower caries counts (PRR= 0.88; 95% CI: 0.56–0.98.56.98). In the univariate analysis, male gender was associated with an approximately 14% higher caries experience (PRR= 1.14; 95% CI: 1.05–1.52), while each additional child increased caries counts by 10% (PRR= 1.10; 95% CI: 1.06–1.89.06.89). Also, refugees who attended dental clinic in the last year had 18% higher caries counts (PRR=1.18, 95% CI: 1.11–1.59.11.59). Conversely, employed participants had significant less caries experience (PRR= 0.83, 95% CI: 0.57–0.86.57.86); however, these factors were only significant in the unadjusted model and did not retain significance in the fully-adjusted model.

**Table 3:** Negative binomial regression analysis with caries experience (DMFT index) as the dependent variable (*N*= 510)

**Variables**		**Unadjusted (Univariate analysis)**		**Adjusted (multivariate analysis)**	
		**PRR (95% C.I)**	**P value**	**PRR (95% C.I)**	**P value**
Gender	Males vs females	1.14 (1.05, 1.52)	0.01*	1.03 (0.86, 1.29)	0.51
Age		1.84 (1.16, 2.94)	<0.001*	1.16 (1.03, 2.34)	<0.001*
Country of origin	Sudan Syria Iraq (Ref)	0.55 (0.17, 1.79) 0.87 (0.27, 3.04) Ref	0.12 0.47 Ref	0.74 (0.53, 1.02) 0.96 (0.68, 1.35) Ref	0.32 0.86 Ref
Marital status	Married vs unmarried	1.11 (0.85, 1.61)	0.25	1.04 (0.92, 1.09)	0.64
Number of children		1.10 (1.06, 1.89)	<0.001*	1.02 (0.94, 1.71)	0.25
Immigration status	With family vs alone	0.61 (0.74, 2.25)	0.37	0.92 (0.67, 2.81)	0.60
Educational level	Illiterate Secondary or less University and higher (Ref)	1.63 (1.24, 2.69) 1.45 (1.05, 1.97) Ref.	<0.001* 0.001* Ref.	1.32 (1.08, 3.49) 1.19 (1.05, 4.32) Ref.	0.001* 0.03* Ref.
Employment status	Employed vs unemployed	0.83 (0.57, 0.86)	0.005*	0.96 (0.84, 1.10)	0.79
Income level	< 3000 vs >3000 EGP	1.29 (1.10, 2.39)	<0.001*	1.06 (1.01, 1.63)	0.04*
Time in Egypt	≤ 5 years vs >5 years	0.65 (0.46, 0.88)	<0.001*	0.90 (0.71, 0.93)	0.02*
Toothbrushing frequency	At least once daily vs irregular	0.71 (0.59, 1.08)	0.18	0.89 (0.75, 1.06)	0.75
Adjunctive dental cleaning	Yes vs no	0.87 (0.83, 1.42)	0.26	0.99 (0.90, 2.31)	0.58
Attended a dental clinic last year	Yes vs no	1.18 (1.11, 1.59)	0.01*	1.09 (0.85, 3.01)	0.39
Access to primary oral healthcare	Yes vs no	0.79 (0.49, 0.95)	<0.001*	0.88 (0.56, 0.98)	0.01*
Frequency of sugary snacking	At least once daily vs irregular	2.48 (1.46, 4.81)	<0.001*	1.39 (1.29, 3.51)	<0.001*
Cigarette smoking	Yes vs no	1.24 (0.36, 3.79)	0.32	1.07 (0.76, 1.24)	0.75
Plaque index		1.59 (1.36, 3.03)	<0.001*	1.46 (1.20, 3.53)	<0.001*

*PRR* prevalence rate ratio, *CI* confidence interval

^*^statistically significant at *P* < 0.05

Variables associated with periodontal disease are shown in table 4. In the multivariate model, the factors associated with increased odds of having periodontal pockets were being male (AOR= 1.87; 95% CI: 1.34–5.17.34.17), older age (AOR = 4.48; 95% CI: 2.68–10.6), lower educational level (illiterate vs university educated, AOR = 5.15; 95% CI: 1.28–17.64.28.64, and secondary educated or less vs university educated, AOR = 2.61; 95% CI: 1.92–14.32.92.32). Behavioral risk factors including daily consumption of sugary snacks (AOR= 2.47; 95% CI: 1.20–6.09) and current smoking status (AOR= 3.18; 95% CI: 1.56–9.12) were also associated with increased periodontal pockets. Furthermore, elevated values of clinical indicators, including Plaque index (AOR= 3.60; 95% CI: 1.92–8.30.92.30), and DMFT index scores (AOR = 2.78; 95% CI: 1.09–6.98.09.98), were associated with increased periodontal pockets. In the univariate analysis, several factors initially showed significant associations that did not persist in the fully-adjusted model. These included country of origin, where participants from Sudan (AOR= 3.61; 95% CI: 2.10–15.21.10.21) and Syria (AOR= 2.93; 95% CI: 1.89–13.92.89.92) had higher odds of periodontal pockets compared to those from Iraq. Also, refugees with low income level (AOR= 1.37, 95% CI:1.05–3.61.05.61), and those who performed adjunctive dental cleaning (AOR= 2.87, 95 CI: 1.38–6.24.38.24) demonstrated an increased risk of periodontal pockets. In contrast, being in Egypt for 5 years or less, brushing teeth at least once daily, and access to primary oral healthcare were associated with lower odds of periodontal pockets (AOR= 0.74, 95% CI: 0.52,−0.94, AOR= 0.80; 95% CI: 1.05–3.23.05.23, and AOR= 0.68; 95% CI: 0.42–0.94.42.94, respectively).

**Table 4:** Binary logistic regression analysis of periodontal disease using CPI index scores (≤2 vs. ≥3) as the dependent variable (*N*= 510)

**Variables**		**Unadjusted (Univariate analysis)**		**Adjusted (multivariate analysis)**	
		**OR (95% C.I)**	**P value**	**OR (95% C.I)**	**P value**
Gender	Males vs females	2.39 (1.61, 4.58)	<0.001*	1.87 (1.34, 5.17)	0.01*
Age		6.93 (3.07, 11.23)	<0.001*	4.48 (2.68, 10.62)	<0.001*
Country of origin	Sudan Syria Iraq (Ref)	3.61 (2.10, 15.21) 2.93 (1.89, 13.92) Ref	<0.001* 0.03* Ref	4.96 (1.29, 19.04) 4.18 (0.98, 17.78) Ref	0.12 0.09 Ref
Marital status	Married vs unmarried	1.92 (0.61, 3.92)	0.40	1.20 (0.81, 5.30)	0.81
Number of children		1.98 (0.82, 6.45)	0.81	140. (0.86, 6.03)	0.49
Immigration status	With family vs alone	0.57 (0.07, 4.19)	0.59	1.19 (0.74, 5.22)	0.87
Educational level	Illiterate Secondary or less University and higher (Ref)	7.29 (1.91, 18.67) 4.32 (1.05, 20.35) Ref.	<0.001* 0.001* Ref.	5.15 (1.28, 17.64) 2.61 (1.92, 14.32) Ref.	0.001* 0.01* Ref.
Employment status	Employed vs unemployed	0.47 (0.33, 4.68)	0.55	0.79 (0.07, 4.20)	0.38
Income level	< 3000 vs >3000 EGP	1.37 (1.05, 3.61)	0.02*	1.18 (0.79, 6.23)	0.41
Time in Egypt	≤ 5 years vs >5 years	0.74 (0.52, 0.94)	0.01*	0.92 (0.82, 1.88)	0.49
Toothbrushing frequency	At least once daily vs irregular	0.80 (1.05, 3.23)	0.004*	0.93 (0.07, 5.03)	0.76
Adjunctive dental cleaning	Yes vs no	2.87 (1.38, 6.24)	<0.001*	1.70 (0.96, 8.31)	0.62
Attended a dental clinic last year	Yes vs no	1.89 (0.97, 6.68)	0.34	1.13 (0.82, 5.11)	0.23
Access to primary oral healthcare	Yes vs no	0.68 (0.42, 0.94)	0.03*	0.92 (0.80, 3.22)	0.08
Frequency of sugary snacking	At least once daily vs irregular	3.61 (1.33, 5.29)	<0.001*	2.47 (1.20, 6.09)	0.01*
Cigarette smoking	Yes vs no	4.01 (1.68, 7.79)	<0.001*	3.18 (1.56, 9.12)	<0.001*
Plaque index		6.91 (1.42, 10.91)	<0.001*	3.60 (1.92, 8.30)	0.03*
DMFT index		3.89 (1.22, 7.32)	<0.001*	2.78 (1.09, 6.98)	0.001*

*OR* odds ratio,*CI* confidence interval

^*^statistically significant at *P* < 0.05

## Discussion

To the best of our knowledge, this is the first cross-sectional study to assess the oral health status of refugees in Egypt, providing an insight on the prevalence of dental caries and periodontal disease among this vulnerable population. The DMFT and CPI indices were used in this study as they represent gold-standard epidemiological tools, which with their widespread use could provide a reliable comparison of our results with global data [28]. Data obtained from the analysis reported, in general, a high prevalence of dental caries and periodontal disease among the examined refugees, and highlighted some sociodemographic, migration-related, and behavioral factors that could be considered as potential risk indicators for these diseases. The study findings showed a high mean DMFT score of 9.25 ± 5.82 among refugees, which aligns with studies conducted among refugee populations [17]. In order to compare our data with a control population, we took into consideration the study by Abbass et al. [31], which evaluated the prevalence of dental caries in Egyptian population among 359 adults aged 18 to 74 years old, reporting a considerably lower mean DMFT score of 6.09 ± 5.7. This highlights the greater oral health challenges faced by refugees, which could be because of the economic stresses they are facing, in addition to the limited healthcare access as evidenced by the high proportion of participants (87.84%) reporting an inability to access or utilize primary oral healthcare services. This limited access to oral healthcare also justifies the high DT component of the caries experience (5.01 ± 3.78) and the low FT component (1.02 ± 2.03), which also suggests that when care is sought for, it is mostly emergency extractions rather than restorative treatments, which is consistent with results reported by a recent systematic review on refugees' oral health [32]. The difficulties faced by refugees in accessing dental health services in the host countries are frequently reported [17, 33] However, when considering Arabic-speaking refugees in Egypt, many of the typical barriers cited in the literature, such as language barriers, significant cultural differences, and lack of health insurance, are less relevant. This is due to the shared language with the local population and the granted access to free healthcare services similar to Egyptian citizens. Hence, we postulate that their limited knowledge and awareness about the dental care system may be a significant, underlying obstacle to utilization. Regarding periodontal health, the mean plaque index score was 1.49 ± 0.68, which again was higher than the average plaque index score of 1.05 ± 0.43 reported for the Egyptian population by Mostafa and El-Refa [34]. As for CPI scores, the majority of refugees (64.51%) exhibited gingivitis (CPI scores 1–2), and a smaller proportion (12.16%) had periodontitis (CPI scores 3–4). Similar values were reported by Lauritano et al.,[35] where only 15% of the migrant population exhibited periodontal pockets, while the majority presented with gingivitis. These values indicate a considerable burden of periodontal disease among the refugee population, which could be attributed to poor oral hygiene practices, limited access to preventive dental care, and socioeconomic challenges. Although a majority of the participants reported brushing their teeth at least once daily (62.35%), the majority still exhibited gingivitis. This could be linked to improper toothbrushing techniques, insufficient duration or force, and lack of knowledge about effective oral hygiene practices, which was reported in several studies [36, 37]. Additionally, the use of unconventional and potentially abrasive adjunctive teeth-cleaning aids, such as charcoal, lemon, and salt, may have contributed to the gingivitis observed among the refugees. These findings highlight the importance of tailoring education programs for the refugees that not only encourage them to brush their teeth but also teach them effective brushing techniques, raise awareness about appropriate oral hygiene tools, and discourage them from using harmful traditional practices such as, charcoal, salt, or lemon.

The regression analysis revealed several factors significantly associated with poor oral health outcomes in this refugee population. Older and less educated individuals showed increased susceptibility to both dental caries and periodontal disease. This pattern is evident not only across diverse non-refugee populations globally [18], but also among refugee populations, such as those in Germany [38]. The association with age could be due to the cumulative nature of oral diseases over time, prolonged exposure to risk factors, and reduced prioritization of oral health, especially since other general health problems tend to increase with age. Lower educational levels, on the other hand, are usually linked to limited health literacy and reduced awareness of preventive oral health measures [39].

Frequent consumption of sugary snacks was significantly associated with the development of both dental caries and periodontal disease. This finding aligns with the results of a recent systematic review that highlighted a trend of increased consumption of high-sugar diets among refugees in host countries compared to their diets in their home countries [40]. Sugar plays a role in caries development by serving as a substrate for acid-producing bacteria, while for periodontal disease it can promote dysbiosis in the oral microbiome and increase inflammation in the gingival tissues [41]. The study findings also showed a significant association between elevated plaque index scores with both outcomes, which agrees with established evidence regarding the important role of plaque in the pathogenesis of both dental caries and periodontal disease [42]. Lower income levels, longer stay in Egypt, and lack of access to primary oral healthcare were significantly associated with a higher DMFT count in both unadjusted and adjusted analyses, but this significant relationship was observed with CPI scores only in the unadjusted analysis. The study by Celeste et al. [43] also reported that income inequality was significantly associated with untreated dental caries, but not with periodontal disease in adjusted models. This highlights the importance of the financial situation of refugees and access to healthcare in attaining preventive services and timely treatment for dental caries, while periodontal disease could be tied more to personal oral hygiene level. The association between longer stay and increased DMFT scores contrasts findings from Saadeh et al. [44], who reported improved oral health conditions with prolonged residence in the United States of America, likely because of better healthcare access over time. This disagreement may reflect the barriers faced by refugees in Egypt, such as limited awareness of available services or difficulties navigating the healthcare system. This is further supported by the significant association between lack of access to primary oral healthcare and higher DMFT scores, suggesting that limited integration into the healthcare system may persist even with longer residence. Thus, targeted awareness campaigns to inform refugees about their oral healthcare entitlements and available clinic locations should be implemented through existing refugee networks and services. Conversely, male gender and being smokers were associated with higher CPI scores in the adjusted analysis, but not with DMFT scores. Many studies reported that males are more susceptible to developing periodontal diseases, which was attributed to factors like differences in oral hygiene practices and lifestyle choices such as, smoking whose effect on oral microbiota and impairing blood flow is well established [45]. Lastly, there was an observed association between dental caries and periodontal disease among the refugees which is consistent with the findings of a recent systematic review [46], which could be explained by shared risk factors for both diseases.

One of the key strengths of this study is that, based on our knowledge, this is the first study in Egypt to assess the oral health of refugees, addressing a significant gap in the existing literature. Furthermore, the inclusion of both oral examinations and questionnaire-based data collection provides a comprehensive approach, allowing for a more detailed and reliable assessment of the oral health status and associated factors within this vulnerable population. However, this study had some limitations. Owing to its cross-sectional design, the current study offered a snapshot of the respondents’ oral health status at a single point in time, while the lack of temporal data limited the ability to establish causal relationships between oral health determinants and measured oral health conditions. Moreover, the snowball sampling could lead to some selection bias; however, as mentioned previously this is the recommended approach for hard-to-reach populations. Another point is the use of a binary (yes/no) question to assess access to oral healthcare, following prior methodology [22] While this effectively quantified the scale of the problem, it did not illuminate the specific barriers responsible. Therefore, our finding that the vast majority of participants lacked access highlights the critical need for future qualitative or mixed-methods research specifically designed to explore these underlying reasons and inform targeted interventions. A further limitation is the use of a partial-mouth examination (index teeth) for both the plaque index and CPI, rather than a full-mouth assessment, which may underestimate the true disease prevalence and severity in entire dentition. However, this approach is a standard protocol recommended by the WHO in large-scale epidemiological surveys to increase efficiency and reduce patient burden [28]. While the use of a self-reported data for the questionnaire allowed the efficient collection of sociodemographic and behavioral data, this method is susceptible to reporting biases, such as recall and social desirability biases. Additionally, the sample was predominantly composed of female respondents, which could potentially be attributed to the fact that women tend to be more communicative and open to sharing their opinions. Another reason could be the nature of the study, which involved oral health examinations, a subject that women are generally more likely to prioritize and engage with due to a greater concern for oral health compared to men. Another limitation of the study was the predominance of Sudanese participants in the sample. This could be attributed to the ongoing Sudan conflict, which has led many individuals to seek support from organizations such as Caritas as they try to overcome the challenges of displacement and secure basic necessities.

## Conclusion

This study highlights the significant oral health problems faced by refugees in Egypt, with high levels of untreated dental caries and periodontal disease. The dominance of decayed over filled teeth, in addition to the low utilization of oral healthcare services, despite the legal entitlements to healthcare, necessitates the need for specifically designed tailored oral health programs for them. These programs should not only address preventing practices but also inform and guide refugees on which oral healthcare services are available and how to access them. The study also identified age, educational and income level, duration of stay in Egypt, frequency of sugary snacks daily, access to primary oral healthcare, and plaque index as factors asscoiated with dental caries. For periodontal disease, the associated factors identified were gender, age, educational level, frequency of sugary snacks, smoking, plaque index, and DMFT index. These factors can aid in tailoring the oral health promotion programs to receive focused preventive regimens and to reduce the burden of oral diseases and improve refugees' oral health.

## Data Availability

The datasets analysed during the current study are available from the corresponding author on reasonable request.

## Abbreviations

IOM: International Organization for Migration
UNHCR: United Nations High Commissioner for Refugees
DMFT: Decayed, Missing, Filled Teeth Index
CPI: Community Periodontal Index
WHO: World Health Organization
